# Surgical treatment of bulbar urethral strictures: tips and tricks

**DOI:** 10.1590/S1677-5538.IBJU.2020.99.04

**Published:** 2020-01-20

**Authors:** Guido Barbagli, Marco Bandini, Sofia Balò, Salvatore Sansalone, Denis Butnaru, Massimo Lazzeri

**Affiliations:** 1 International Center for Reconstructive Urethral Surgery Arezzo Italy International Center for Reconstructive Urethral Surgery, Arezzo, Italy;; 2 Urological Research Institute San Raffaele Hospital Vita-Salute San Raffaele University Milan Italy Unit of Urology, Urological Research Institute (URI), San Raffaele Hospital, Vita-Salute San Raffaele University, Milan, Italy;; 3 Department of Experimental Medicine and Surgery University of Tor Vergata Rome Italy Department of Experimental Medicine and Surgery, University of Tor Vergata, Rome, Italy;; 4 Institute for Regenerative Medicine Moscow State Medical University Moscow Russia Institute for Regenerative Medicine, Sechenov First Moscow State Medical University, Moscow, Russia;; 5 Department of Urology Istituto Clinico Humanitas IRCCS Clinical and Research Hospital Milan Italy Department of Urology, Istituto Clinico Humanitas IRCCS, Clinical and Research Hospital (ML), Rozzano, Milan, Italy

**Keywords:** Urethra, Anastomosis, Surgical, Surgical Procedures, Operative

## Abstract

The surgical treatment of bulbar urethral strictures is still one of the most challenging reconstructive-surgery problems. Bulbar urethral strictures are usually categorized as traumatic and non-traumatic strictures depending on the aetiology. The traumatic strictures are caused by trauma and they determine disruption of the urethra with obliteration of the urethral lumen, ending with fibrotic gaps between the urethral ends. Differently, the non-traumatic urethral strictures are mainly caused by catheterization, instrumentation, and infection, or they can also be idiopathic. They are usually associated with spongiofibrosis of the segment of the urethra that has been involved. Worldwide, two different surgical approaches are currently adopted for bulbar urethral repair: transecting techniques with end-to-end anastomosis and non-transecting techniques followed by grafting. Traumatic obliterated strictures require transection of the urethra allowing complete removal of the fibrotic tissue that involves the urethral ends. Conversely, non-traumatic, non-obliterated urethral strictures require augmentation of the urethral plate using oral mucosa grafts. Nowadays, it is still difficult to choose the correct surgical management for non-obliterated bulbar stricture repair. Indeed, different surgical techniques have been proposed (pedicled flap vs free graft, dorsal vs ventral placement of the graft, non-transecting technique using or non-using free graft, etc.) but none emerged as the best solution since all techniques have showed similar success and complication rates. Consequently, the final choice is still based on surgeon’s preferences and patient’s characteristics. Within the current manuscript, we like to present some of our tips and tricks that we developed along our prolonged surgical experience on the treatment of bulbar urethral strictures. These might be of interest for surgeons that approach this complex surgery. Moreover, our suggestions want to be useful regardless the type of chosen technique being adaptable for different scenario.

## INTRODUCTION

The treatment of bulbar urethral strictures using end-to-end anastomosis was firstly described in 1914 by Hamilton Russell from Melburne, Australia. Across the years, many authors reported excellent results using excision of urethral strictures and end-to-end anastomosis, with some innovative technical suggestions ( 1 - 7 ). In 2007, we reported our case series of 153 treated patients that received bulbar end-to-end anastomosis. Between those patients, complications were modest with 14 (23.3%) patients that experienced ejaculatory dysfunction, 11 (18.3%) had decreased glans sensitivity, 7 (11.6%) had the gland neither full or swollen during erection, 1 (1.6%) had a cold gland during erection ( 8 ). The scenario of urethral stricture repair was further mutated in 2011, when Andrich and Mundy described a new technique: the non-transecting anastomotic bulbar urethroplasty. Here, the corpus spongiosum and the urethral arteries were not transacted during the procedure. Thanks to the blood supply preservation, the authors described absence of any sort of sexual complications at short and long-term ( 9 ). Nowadays, the choice between transecting (end-to-end) and non-transecting (free graft one-stage urethroplasty) techniques is still controversial ( 10 , 11 ), and yet none of the two techniques has prevailed over the other.

The grafting era of reconstructive bulbar urethral stricture repair started in 1996 when two fundamental techniques were described. Morey and McAninch presented the technique for harvesting the oral mucosal graft from the cheek and the ventral grafting of the urethra ( 12 ). Additionally, Barbagli et al. described for the first time the dorsal grafting of the urethra ( 13 ) with buccal mucosa. These two different techniques were further described by Barbagli et al. in 2011 and 2012 ( 14 , 15 ), as well as by many different authors with similar or modified approaches ( 16 - 23 ). Both have largely contributed to improve surgical outcomes in patients treated for bulbar strictures.

The aim of this narrative review is to describe some tips and tricks, as well as useful steps in performing any type of bulbar urethroplasty. Understanding the peri and intra-operative challenges that may lead to better urethroplasty performance with higher satisfaction rate for surgeons and their patients. We included in this review many drawings and intraoperative photos that can be used as examples for the reader to better understand our practice.

## MAIN TEXT

### Selection of the surgical technique

The appropriate selection of the surgical technique is mainly based on patient’s and stricture’s characteristics.

### Patient features

**Age:** Older patients are preferred candidates for end-to-end anastomosis instead of graft augmentation. We say that because operating time is shorter, quality of the buccal mucosa graft might not be as good as in young patients, and also because potential adverse sexual events may have a marginal impact on the quality of life of elderly men. In young patients instead, the bulbar urethroplasty should not be a cause of any sexual or ejaculatory dysfunction. In consequence, graft augmentation is usually preferred. Additionally, for the proximal bulbar urethra, the use of the ventral grafting is more safe than the dorsal counterpart. Indeed, during the ventral approach, the urethral dissection is limited to the ventral surface away from vessels and functional nerves. Conversely, aggressive dissection is required using the dorsal approach, with associated higher risk of sexual impairment.

**BMI:** Obese patients are no ideal candidates for dorsal grafting. Here, the deepest and fatty perineum may render very difficult to access to the dorsal urethral surface, especially for the proximal urethra, increasing the risk of bleeding and subsequent sexual dysfunction.

**Previous surgery:** In patients with previous hypospadias repair or penile surgery, the retrograde blood supply to the bulbar urethra may be greatly compromised or absent. Thus, the complete transection of the bulbar urethra (and its anterograde blood supply from the bulbar arteries) may cause a bulbar urethral necrosis ending in early stricture recurrence. This should be kept in mind every time we operate these complex patients.

### Stricture features

**Aetiology:** Bulbar strictures related to previous blunt perineal trauma with urethra disruption require end-to-end anastomosis.

**Figure 1:**
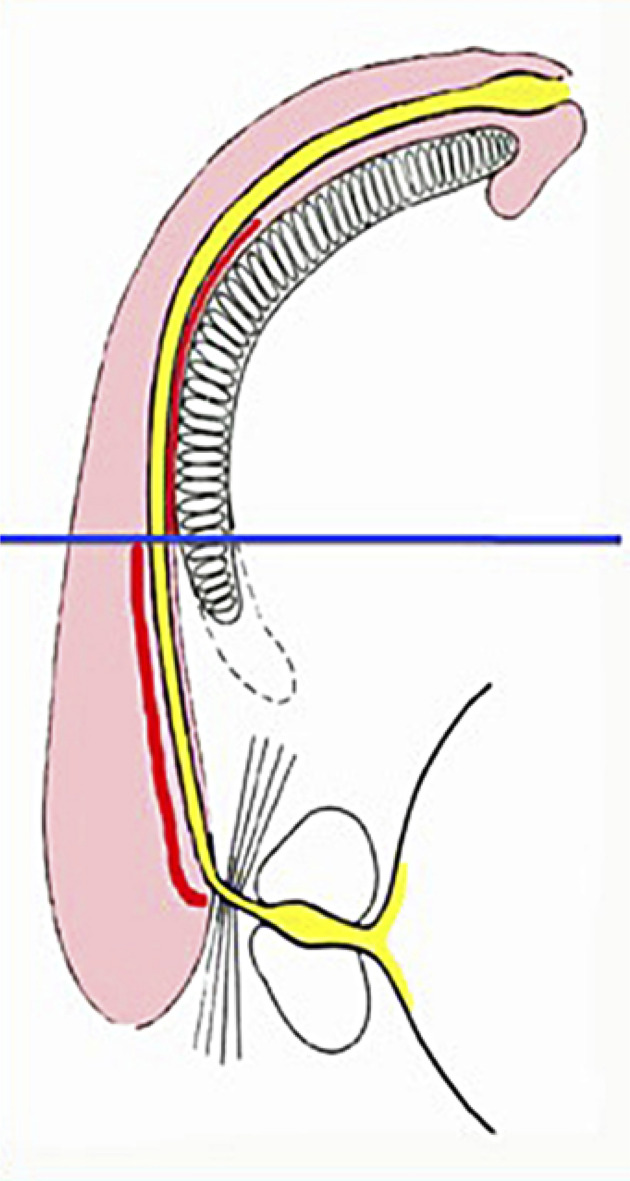
The different location of the graft (in red) according to the thickness of the spongiosum tissue: dorsal location on the distal bulbar urethra, ventral location on the proximal bulbar urethra.

**Site:** In distal peno-bulbar strictures, the end-to-end anastomosis may cause penile cordee and/or sexual dysfunction, and it is consequently not the preferred technique for these types of strictures. Grafting techniques should be instead preferred. However, from the distal bulbar urethra up to the tip of the penis, the spongiosum tissue is thin and does not provide adequate support for a ventral graft. For these reasons, it is better to use the dorsal onlay approach, and to reserve the ventral approach only for the proximal part of the bulbar urethra, where more abundant spongiosum tissue can supply the graft ( Figure-1 ).

**Length:** Strictures up to 2 cm are ideal for end-to-end anastomosis. In longer strictures, complete transection of the urethra and subsequent removal of the scarred tissue may create unexpected loss of tissue and longer gap between the two urethral ends. In these situations, end-to-end anastomosis are not recommended since they cannot provide tension-free anastomosis, with consequent higher risk of recurrent stricture. Planning the end-to-end urethroplasty, surgeons need to be mindful that urethrography may underestimate the real stricture length. Moreover, when they perform an end-to-end anastomosis, both the urethral ends should be spatulated for approximately one cm on each side. In consequence, 1 cm stricture requires the removal of 3 cm of urethra shortening considerably the urethra.

### Tips and tricks for bulbar urethroplasty

We like present also some important and useful suggestions to render the surgery safest for the patient and easier for the surgeon.

### Preparation of the patient for surgery

**Figure 2:**
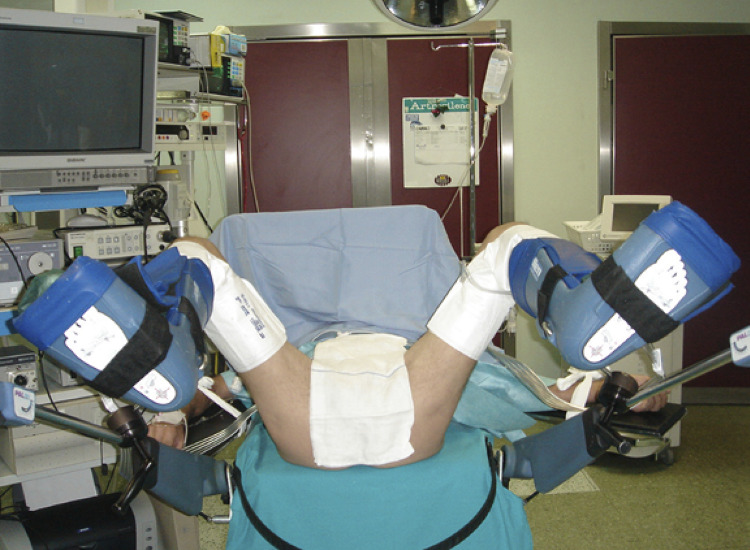
Simple lithotomy position using Allen stirrups and sequential inflatable compression sleeves.

For any bulbar urethroplasty, simple or complex, we suggest to rely on the simple lithotomy position using the Allen stirrups ( Figure-2 ). This might avoid any compression on the popliteal fossa that can cause compartmental syndrome or neuro-muscular problems.

The use of sequential inflatable compression sleeves ( Figure-2 ), greatly reduces the risk of vascular problems to the legs and embolism. Furthermore, the use of these devices is comfortable for the patients during the postoperative recovery because they facilitate the relaxation of the muscles of the lower limbs. For any kind of urethroplasty, we kindly ask to the anaesthesiologist to perform general anaesthesia (no epidural anaesthesia) with controlled hypotension (range 90mmhg - 40mmhg). This suggestion is crucial to avoid bleeding.

### Preparation of the urethra for surgeon

**Figure 3:**
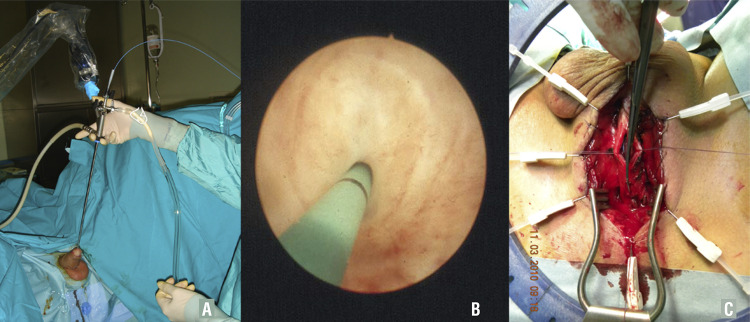
– A) Urethroscopy is performed using 7F instrument; B) The 3F guidewire is inserted through the stricture; C) Following the guidewire, the urethral opening is more faster, easier, and sure.

Before starting the bulbar urethroplasty we suggest to insert 3Fr guidewire through the urethra ( Figures 3A and 3B ). The guidewire is an important suggestion to avoid any problem during surgery, especially to avoid the risk of losing the proximal urethral lumen. Following the guidewire ( Figure-3C ), the urethral opening is faster, easier and of course safer.

### Harvesting the oral mucosal graft

**Figure 4:**
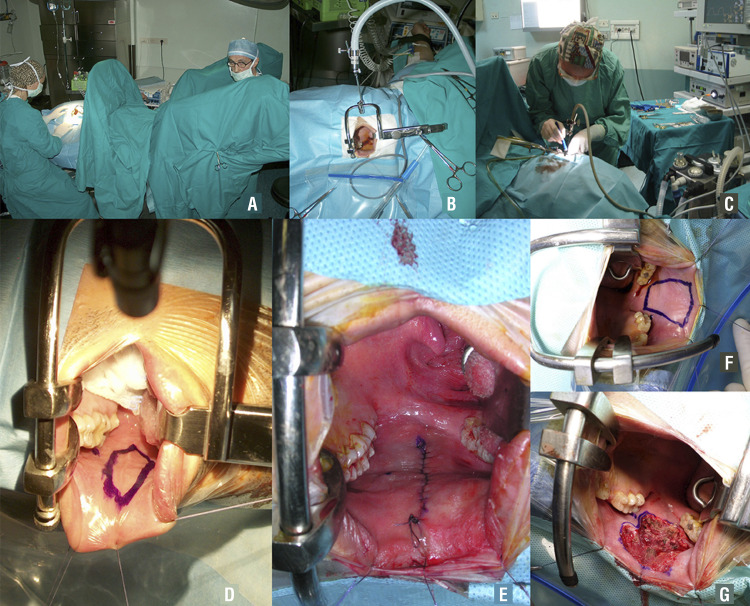
A) The double team; B) The Kilner-Doughty mouth retractor in place; C) The assistant harvesting the graft; D) Ovoidal shape graft for one-stage urethroplasty; E) Closure of the harvesting site; F) Rectangular shape graft for two stage urethroplasty; G) Non-closure of the harvesting site.

As suggested by Morey and McAninch in 1996 ( 12 ), to harvest the oral mucosal graft, we also rely on a double team. The first one can harvest the buccal mucosa graft, while the second team can carry the urethral dissection and preparation ( Figure-4A ). The use of the double team reduces the operative time, the risk of cross-contamination during surgery, and it is a good opportunity for young residents to start their training in reconstructive urethral surgery, taking care of the harvesting part of the buccal mucosa graft. In our daily practice, the cheek represents the preferred site for harvesting the graft. A Kilner-Doughty mouth retractor is placed in situ ( Figure-4B ), and using this retractor only one assistant is required for the harvesting procedure ( Figure-4C ). For one-stage urethroplasty, we harvest an ovoidal oral mucosal graft ( Figure-4D ), and we always close the harvesting site ( Figure-4E ). For 2-stage urethroplasty, we harvest a rectangular graft ( Figure-4F ), and we don’t close the harvesting site ( Figure-4G ). Using these techniques, we reported a low incidence of early and late post-operative complications or sequelae, but high patient’s satisfaction, as reported in a series of 553 patients ( 24 ).

### The true anatomy of the proximal bulbar urethra

**Figure 5:**
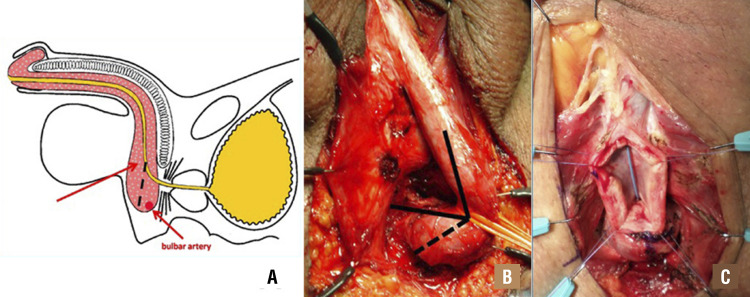
– A) The urethra don’t progress downward but heading straight to the bladder; B) The true direction of the proximal bulbar urethra; C) The urethra is ventrally opened and the stricture is evident.

**Figure 6:**
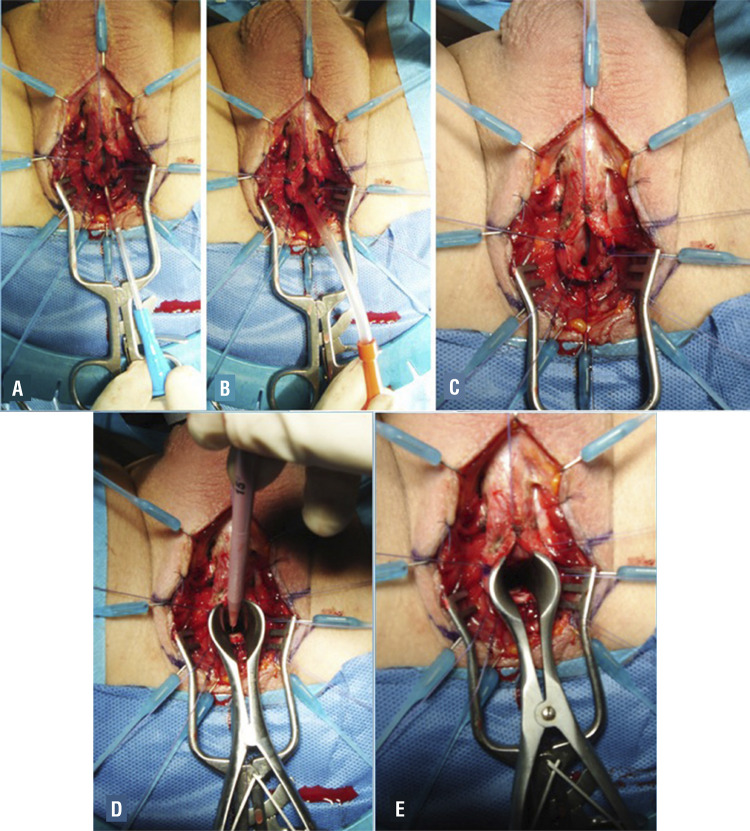
– A-E) Progressive urethral dilation over catheter until 16F.

To know well the anatomy of the proximal bulbar urethra is fundamental to whom who want to perform urethral surgery. In the proximal part of the bulbar urethra, the urethral tube does not progress downward inside the spongiosum tissue, but it heads straight to the bladder ( Figures 5A and 5B ). Thus, when we expose the distal part of the urethral stricture ( Figure-5C ), it is not necessary to open the spongiosum tissue for the last 3 cm, since the urethra has already turned into the perineum. This approach might also avoid excessive bleeding because it spares the bulbar arteries ( Figure-5A ). When we approach the urethral lumen, we usually make progressive dilations of the stricture ( Figures 6A-C ), until 16 Fr. At this point, we check if a nasal speculum can be inserted in the proximal part of the urethra and subsequently we enlarge the proximal lumen by making several incisions of the scarring tissue at 6 o’clock ( Figure-6D ). We repeat these steps until the speculum can be widely opened ( Figure-6E ) inside the proximal urethra.

### Anastomosis of the oral graft to proximal urethral mucosa

**Figure 7:**
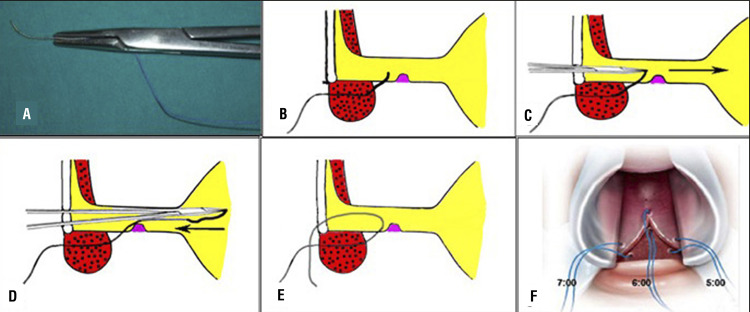
– A) The j-shape needle; B) The needle is moved in front up to the verumontanu; C) The needle is pushed head into the bladder; D and E) The needle is withdrawing back; F) Three stitches are inserted at 5, 6, 7 o'clock near the veru montanu.

**Figure 8:**
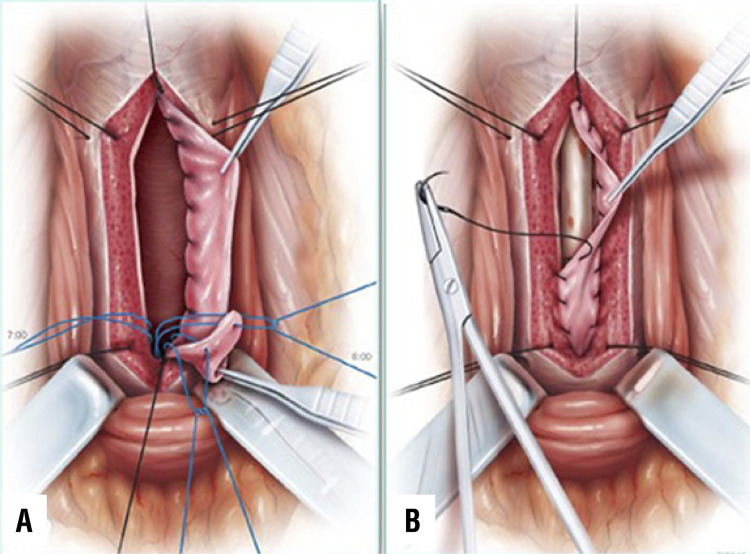
– A) The 3 stitches are inserted into the proximal end of the graft; B) The graft is moved near the veru montanu.

During ventral onlay graft urethroplasty, it is mandatory to perform the anastomosis between the oral graft and the urethral mucosa as proximal as possible, just in front of the verumontanum. This trick is crucial if we want to avoid recurrence of the stricture on the proximal tract of the anastomosis. Using a 4/0 Vicryl, with the needle modified into a J shape ( Figure-7A ), we pass the stich, from outside to inside, through the spongiosum tissue until the verumontanum ( Figure-7B ). Subsequently, the tip of the needle is pushed head toward the bladder ( Figure-7C ) and withdrew backward outside the urethra ( Figures 7D and E ). By using this technique, 3 stitches are inserted at 5, 6, 7 o’clock positions near the verumontanum ( Figure-7F ). The stitches are then passed through the proximal end of the oral graft ( Figure-8A ), and when they are tied up, the oral graft is moved towards the verumontanum ( Figure-8B ), filling the gap.

## CONCLUSIONS

Within the current review we reported many tips and tricks that we developed, over the past years, and that we have progressively integrated in our daily practice. These suggestions have been used for any type of bulbar urethroplasty, resulting in shorter surgical time and lower incidence of post-operative complications. Taken together, the experience that we have maturated over these years has increased the safety and the success rate of our urethroplasties. Noteworthy, the choice of the surgical technique is still a surgeon choice, rather than a “guideline recommended” approach. Surgeons should always take into account patient’s (age, BMI, previous surgery) characteristics and stricture’s (aetiology, site, length) features before choosing the appropriate technique. The available literature provides many reports about different techniques, but we believe that the surgical experience, as well as surgical preference and background still represent the most important factors that should influence the choice of the correct approach. We hope that our suggestions might help surgeons to improve their daily surgical practice.
